# Weather-Related Variations of Mycotoxins in Maize: A 2024 Study from AP Vojvodina (Serbia) and the Republic of Srpska (Bosnia and Herzegovina)

**DOI:** 10.3390/foods15142508

**Published:** 2026-07-15

**Authors:** Elizabet Janić Hajnal, Milan Vukić, Ivana Bogić, Lato Pezo, Milorad Miljić, Ivica Đalović

**Affiliations:** 1 Institute of Food Technology, University of Novi Sad, Bulevar cara Lazara 1, 21000 Novi Sad, Serbia; milorad.miljic@fins.uns.ac.rs; 2 Faculty of Technology Zvornik, University of East Sarajevo, Karakaj 34A, 75400 Zvornik, Bosnia and Herzegovina; milan.vukic@tfzv.ues.rs.ba; 3 Faculty of Sciences, University of Novi Sad, Trg Dositeja Obradovića 3, 21102 Novi Sad, Serbia; ivana.b3556@gmail.com; 4 Institute of General and Physical Chemistry, University of Belgrade, Studentski Trg 12-16, 11000 Belgrade, Serbia; latopezo@yahoo.co.uk; 5 Institute of Field and Vegetable Crops, Maksima Gorkog 30, 21000 Novi Sad, Serbia; ivica.djalovic@ifvcns.ns.ac.rs

**Keywords:** maize, LC-MS/MS, regulated mycotoxins, non-regulated mycotoxins, occurrence, weather conditions

## Abstract

Climate variability strongly affects fungal ecology and mycotoxin contamination patterns in maize. This study evaluated the influence of weather conditions during the 2024 growing season on the occurrence and co-occurrence of regulated and emerging mycotoxins in maize from two Southeast European regions: the Autonomous Province of Vojvodina (APV), Serbia, and the Republic of Srpska (RoS), Bosnia and Herzegovina (BIH). A total of 266 maize samples were analysed using an LC-MS/MS method targeting 21 fungal metabolites. The exceptionally hot and dry summer conditions, particularly the severe drought recorded in APV during August, were associated with regional differences in mycotoxin profiles. APV maize showed higher contamination with drought-related mycotoxins, especially aflatoxins, while fumonisins were highly prevalent in both regions. Overall, fumonisins were the dominant regulated mycotoxins (88.0%), followed by aflatoxins (41.4%) and T-2/HT-2 toxins (31.6%), whereas deoxynivalenol and zearalenone occurred less frequently. Emerging metabolites showed high prevalence, particularly alternariol monomethyl ether (86.5%), moniliformin (74.4%), and ergot alkaloids (53.4%). Co-occurrence analysis revealed 189 different combinations, with up to ten mycotoxins detected simultaneously. These findings demonstrate region-specific weather-driven shifts in maize contamination and emphasize the need for multi-mycotoxin monitoring strategies including both regulated and emerging contaminants.

## 1. Introduction

Maize (*Zea mays* L.) is one of the most significant cereal crops globally, serving as a primary component in food, livestock feed, and industrial applications. In the Balkan region, particularly in the Republic of Serbia and Bosnia and Herzegovina (BIH), maize production is of vital economic importance, contributing substantially to agricultural gross domestic product (GDP) and supporting a robust export sector [1,2]. However, the safety and quality of maize are perpetually threatened by mycotoxins—toxic secondary metabolites produced by various filamentous fungi, primarily from the *Aspergillus*, *Fusarium*, and *Penicillium* genera [3]. Mycotoxin contamination poses severe health risks to humans and animals, including carcinogenic, immunosuppressive, and nephrotoxic effects, while simultaneously causing significant economic losses through crop rejection and decreased livestock productivity [4,5].

The occurrence and concentration of mycotoxins in maize are strongly influenced by environmental factors, with weather conditions during the growing and harvesting seasons being the most critical drivers [6]. In particular, temperature and precipitation patterns dictate the competitive advantage of specific fungal species. For instance, hot and dry conditions, often associated with drought stress, favor the proliferation of *Aspergillus flavus* and the subsequent production of aflatoxins (AFs) [7,8]. Conversely, high humidity and moderate temperatures during the flowering stage (silking) typically promote the growth of *Fusarium* species, leading to the accumulation of deoxynivalenol (DON) and zearalenone (ZEA) [9]. Fumonisins (FBs), produced by *F. verticillioides*, are frequently detected across a wider range of conditions in the Balkans but often peak in years with alternating dry and wet periods [10,11].

Besides traditionally regulated mycotoxins, increasing attention has recently been directed toward so-called emerging and non-regulated fungal metabolites, whose occurrence in maize may represent an underestimated food and feed safety issue. Among them, moniliformin (MON), *Alternaria* toxins, and ergot alkaloids (EAs) have gained particular interest due to their frequent detection, possible co-occurrence with regulated mycotoxins, and still limited toxicological and regulatory data [12,13,14]. MON, mainly produced by *F. proliferatum* and *F. subglutinans*, has been increasingly reported in maize from Serbia and neighbouring regions. A recent four-year survey of Serbian maize demonstrated a high prevalence of MON and confirmed that its occurrence is strongly affected by annual weather variability, particularly temperature and precipitation patterns during the maize growing season [11].

In addition to *Fusarium* metabolites, *Alternaria* toxins, including alternariol (AOH), alternariol monomethyl ether (AME), and tentoxin (TEN), are increasingly recognized as relevant emerging contaminants of cereals and cereal-based products due to their frequent occurrence and potential toxicological significance [15,16,17]. Although no maximum permitted levels have yet been established for these compounds, their frequent occurrence and potential toxicological effects have led the European Commission to recommend increased monitoring of *Alternaria* toxins in food commodities [18]. Similarly, EAs, traditionally associated with C*laviceps* contamination of small-grain cereals, have recently been included in multi-mycotoxin monitoring approaches due to their possible occurrence in various cereal-based matrices [14,19]. The increasing detection of these emerging metabolites indicates that climate-driven changes may alter fungal populations and expand the spectrum of mycotoxins occurring in maize [20].

These general relationships are well documented in the Balkan region, where recent studies have demonstrated that climate change has substantially altered mycotoxin contamination patterns, particularly in Serbia, Croatia, Romania, and across the wider Balkan region. Increasing temperatures, prolonged droughts, and irregular precipitation have been identified as the principal drivers shaping fungal populations and mycotoxin occurrence in cereals, with hot and dry conditions favoring aflatoxin contamination, whereas wetter growing seasons promote the accumulation of *Fusarium* mycotoxins, including deoxynivalenol (DON) and zearalenone (ZEA) [21,22,23,24,25,26,27,28]. Fumonisins remain consistently prevalent in maize throughout the region regardless of annual climatic variability, while aflatoxins exhibit pronounced year-to-year fluctuations associated with extreme drought events [21,22,23,24,25,29]. In addition to regulated mycotoxins, increasing attention has been directed towards emerging fungal metabolites, particularly moniliformin (MON), beauvericin (BEA), enniatins (ENs), fusaproliferin (FUS), and minor *Aspergillus* and *Penicillium* metabolites, whose occurrence has been documented in Serbia and Albania and shown to be strongly influenced by weather conditions [24,30]. Nevertheless, comprehensive LC-MS/MS-based studies simultaneously covering regulated and emerging mycotoxins remain limited for some parts of the Balkan region. This gap is particularly evident for maize originating from the Republic of Srpska, Bosnia and Herzegovina, where available data are scarce and mainly focused on a narrower spectrum of *Fusarium* or regulated mycotoxins.Within this regional context, the Autonomous Province of Vojvodina (APV), Serbia, and the Republic of Srpska (RoS), Bosnia and Herzegovina (BIH) have experienced a noticeable shift toward a warmer and more arid climate, characterized by more frequent and intense extreme weather events [20,31]. Historical data from these regions reveal a close association between climatic variability and changes in mycotoxin contamination profiles. The devastating drought of 2012, for example, triggered a major aflatoxin crisis in the region, which had previously been considered a moderate-to-low risk zone for these toxins [32]. Similar patterns of elevated mycotoxin risk were observed during the hot and dry seasons of 2013, 2015, and 2021 [33,34,35]. These recurring events underscore the necessity for continuous monitoring and the development of predictive models based on meteorological data to safeguard regional food and feed safety [36]. The 2024 growing season in the Balkans was marked by exceptionally severe meteorological conditions. Following an unusually warm spring, the region was subjected to a series of record-breaking heatwaves and a prolonged drought during the critical summer months of July and August. In parts of Vojvodina and the Republic of Srpska, temperatures frequently exceeded 40 °C, and some agricultural areas recorded periods of over 40 consecutive days without significant precipitation [37]. These conditions not only stunted crop development—with expected yield reductions of up to 20%—but also created a high-risk environment for thermophilic fungal species and the associated accumulation of mycotoxins [38,39].

Considering the increasing impact of climate variability on fungal ecology and the limited availability of comprehensive multi-mycotoxin data for maize from BIH, particularly the RoS, this study was designed to comprehensively assess maize contamination during the exceptionally hot and dry 2024 growing season. Maize samples collected from two important agricultural regions in Southeast Europe, the APV, Serbia, and RoS, BIH, were investigated to determine how regional differences in temperature and precipitation patterns influenced the occurrence, concentration levels, and co-occurrence of fungal metabolites. Using a multi-mycotoxin LC-MS/MS approach, the study covered a broad spectrum of regulated mycotoxins and emerging/non-regulated fungal metabolites, enabling a comprehensive assessment of contamination profiles beyond conventional monitoring strategies. The obtained results aim to improve understanding of climate-related shifts in mycotoxin patterns and support the development of more effective monitoring and risk management approaches for maize safety in the region. To the best of our knowledge, this study represents the first comprehensive LC-MS/MS-based characterization of both regulated and emerging mycotoxins in maize originating from the RoS (BIH).

## 2. Materials and Methods

### 2.1. Samples

**Figure 1:**
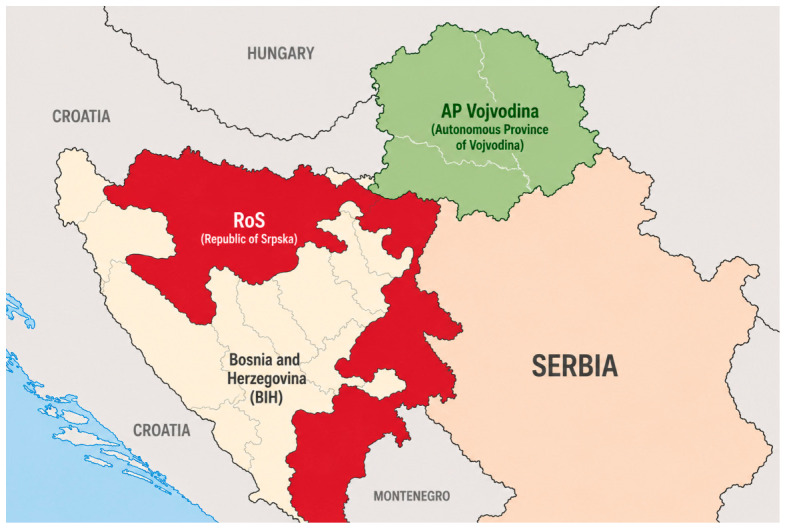
Regions of maize sampling in AP Vojvodina (Serbia) and RoS (Bosnia and Herzegovina).

A total of 85 maize samples were randomly collected during September 2024 from the main maize-producing areas of Northern Serbia (Autonomous Province of Vojvodina, APV; latitude 45°18′ N, longitude 20°09′ E, and altitude 111 m). In Northern Bosnia and Herzegovina (BIH), specifically the Republic of Srpska (RoS; latitude 44°50′ N, longitude 17°55′ E, and altitude 150 m), 181 maize samples were collected during September and October 2024 (Figure 1). The difference in sample numbers between APV (*n* = 85) and the RoS (*n* = 181) reflects differences in sample availability rather than an intentional sampling design. To investigate the impact of weather conditions on mycotoxin occurrence in maize and to minimize the influence of potential secondary contamination during storage, samples from both regions were collected immediately after harvest or directly from drying facilities, prior to further storage in silos and distribution. Sampling in both countries was performed in accordance with Commission Implementing Regulation (EU) 2023/2782 [40] for the control of mycotoxins in food and feed. After collection, maize samples from the RoS were transported to the Institute of Food Technology in Novi Sad during October–November 2024 for further mycotoxin analysis.

Representative maize samples from both regions were prepared from approximately 10 kg aggregate samples at the Institute of Food Technology in Novi Sad. Sample preparation included homogenization (Nauta mixer, model 19387, Nauta Patenten, Haarlem, The Netherlands), quartering, milling (Knifetec™ 1095 mill, Foss, Höganäs, Sweden), packing into zip-lock bags (500–600 g), and storage at −18 °C until further analysis. At the beginning of December 2024, laboratory samples were removed from frozen storage and re-homogenized using a rotary laboratory mixer (RRM Mini-II, Ludwigshafen, Germany) prior to analysis by liquid chromatography–tandem mass spectrometry (LC-MS/MS).

### 2.2. Weather Analysis

To investigate the influence of weather conditions on the natural occurrence of the examined mycotoxins, a detailed analysis of meteorological parameters during the maize growing season (April–October) of 2024 was performed for the investigated regions in Serbia and BIH. Meteorological data, including monthly average air temperature and total precipitation, were obtained from the Republic Hydrometeorological Service of Serbia [37] and the Republic Hydrometeorological Service of the Republic of Srpska [41,42]. Deviations in temperature and precipitation recorded during the 2024 growing season were evaluated by comparison with long-term average values calculated for the reference periods 1991–2020 for APV and 1990–2020 for RoS, respectively.

### 2.3. LC-MS/MS Analysis of Mycotoxins

The quantification of mycotoxins in maize samples was carried out using a modified liquid chromatography–tandem mass spectrometry (LC-MS/MS) method adapted from the protocol reported by Hofmann and Scheibner [43]. The applied analytical method enabled the simultaneous detection and quantification of 21 mycotoxins. LC-MS/MS-grade methanol (MeOH) and water (Carlo Erba, Val de Reuil, France), formic acid (Fluka Analytical, Sigma-Aldrich, Steinheim, Germany), and HPLC-grade acetonitrile (ACN) (Fisher Scientific, Geel, Belgium) were used for sample preparation and chromatographic analysis. The T-2 toxin reference standard (100 µg/mL) was obtained from LGC Standards, while other analytical standards were purchased from Fluka Analytical and Romer Labs. Standards of deoxynivalenol (DON), fumonisins (FB1 and FB2), HT-2 toxin, zearalenone (ZEA), tentoxin (TEN), alternariol (AOH), and alternariol monomethyl ether (AME) were supplied by Fluka Analytical, whereas moniliformin (MON), the six-component EAs mixture, and the mixed aflatoxin standard were obtained from Romer Labs. A multi-mycotoxin stock solution containing 21 standards was prepared using MeOH/water (50/50, *v*/*v*). Matrix-matched calibration standards were prepared by fortifying blank maize extracts with appropriate concentrations of the standard mixture. Before preparation, individual standards were evaporated under controlled conditions and reconstituted in MeOH/water (50/50, *v*/*v*). Quantitative analysis was performed using eleven calibration points within the concentration range of 0.01–200 µg/kg. The calibration curves demonstrated good linear responses for all investigated analytes, with coefficients of determination (*r*^2^) exceeding 0.9915. All individual standards and prepared stock solutions (10 μg/mL) were stored at −18 °C until use.

#### 2.3.1. Sample Preparation

Sample preparation prior to LC-MS/MS analysis was performed according to the method described by Hofmann and Scheibner [43], with minor modifications. HPLC-grade acetonitrile (Fisher Scientific, Geel, Belgium) and purified water obtained using an Adrona Water Purification system (Riga, Latvia) were used for the extraction procedure. Briefly, 5 g of finely ground maize sample was weighed into a 50 mL polypropylene centrifuge tube and extracted using 20 mL of acetonitrile/water mixture (80/20, *v*/*v*). The extraction was carried out by shaking the samples on a horizontal shaker (Biosan, Latvia) at 350 rpm for 60 min, followed by centrifugation (Boeco, Hamburg, Germany) at 4000 rpm for 5 min. Subsequently, 400 µL of the obtained supernatant was mixed with 600 µL of MeOH/water (50/50, *v*/*v*), and the final extract was passed through a 0.2 µm polytetrafluoroethylene (PTFE) syringe filter into LC-MS/MS vials prior to analysis.

#### 2.3.2. LC-MS/MS Analysis

Mycotoxin detection and quantification were performed using a Vanquish Core HPLC system coupled with a TSQ Quantis Triple Quadrupole mass spectrometer equipped with a heated electrospray ionization (HESI) interface (Thermo Fisher Scientific, Waltham, MA, USA). Chromatographic separation was achieved on a Hypersil GOLD C18 Selectivity HPLC column (100 × 2.1 mm i.d., 1.9 μm particle size). The LC-MS/MS analysis was conducted according to Thermo Fisher Scientific Application Note 65969 [43], with certain modifications. Briefly, chromatographic separation was performed using a binary mobile phase consisting of water and methanol, both supplemented with 0.1% formic acid, with initial proportions of 95% water and 5% methanol. The autosampler and column temperatures were maintained at 20 °C and 40 °C, respectively, while the injection volume was set to 10 μL. The complete LC-MS/MS acquisition parameters, including retention times (RT), ionization polarity, precursor ions (*m*/*z*), quantifier and qualifier ions (*m*/*z*), and the corresponding collision energies (CE) for all target analytes have been previously reported by Živančev et al. [44]. Nitrogen was applied as the sheath, auxiliary, and sweep gas at flow settings of 30, 6, and 1 arbitrary units (Arb), respectively, whereas argon was used as the collision-induced dissociation (CID) gas at a pressure of 1.5 mTorr. The ion transfer tube temperature was maintained at 325 °C, and the vaporizer temperature was set at 350 °C. The total cycle time was 0.5 s. Instrument control, data acquisition, and processing were performed using TSQ Quantis 3.2 Tune and TraceFinder 5.1 software (Thermo Fisher Scientific, Waltham, MA, USA).

#### 2.3.3. LC-MS/MS Method Performance

Method validation was performed according to the recommendations described in the Technical Report CEN/TR 16059 [45], Commission Implementing Regulation (EU) 2023/2782 [40], and to the European Commission SANTE/11312/2021 analytical quality control guidelines [46]. The performance characteristics of the applied LC-MS/MS method were evaluated by assessing linearity, recovery, precision (repeatability and within-laboratory reproducibility), limits of detection (LOD) and quantification (LOQ), and matrix effects. Matrix effects were evaluated through signal suppression/enhancement (SSE), calculated as the ratio between the slope of the matrix-matched calibration curve and the slope of the solvent-based calibration curve (Table 1). Extraction recovery (R_E_, %) was calculated by comparing the slope obtained from the spiked sample calibration curve with the slope of the matrix-matched calibration curve (Table 1). Method precision was assessed for all target mycotoxins by analysing blank maize samples fortified at six concentration levels, with six replicate measurements performed at each level (Table S1). Repeatability and within-laboratory reproducibility were expressed as relative standard deviations (RSDr and RSDR, respectively). The recoveries of all target mycotoxins (73.3–114%) complied with the performance criteria specified in the applied validation guidelines (Table 1), while the precision, expressed as RSDr and RSDR, ranged from 2.15 to 25.5% and 2.53 to 28.5%, respectively, depending on the analyte and spiking level (Table S1). LOD and LOQ values were estimated by analysing decreasing concentrations of matrix-matched standards and were defined as the lowest concentrations providing signal-to-noise (S/N) ratios of ≥3 and ≥10, respectively.

The obtained validation parameters, including recovery, precision, matrix effects, linearity, LOD, and LOQ values (Table 1 and Table S1), fulfilled the recommended performance criteria, confirming the suitability of the LC-MS/MS method for reliable multi-mycotoxin determination in unprocessed maize samples.

### 2.4. Statistical Analysis

Statistical analysis of the mycotoxin concentration data was performed using TIBCO Statistica^®^ software, version 14.0.15 [47]. Descriptive statistical parameters, including mean, standard deviation, median, minimum, maximum, variance, and sample count, were calculated for each mycotoxin in samples originating from RoS (BIH) and the APV (Serbia). Differences in mycotoxin concentrations between the two geographical regions were evaluated using one-way analysis of variance (ANOVA). The *F*-statistic and corresponding *p*-value were calculated for each analyte, and statistical significance was accepted at *p* < 0.05.

## 3. Results and Discussion

### 3.1. Weather Condition Analysis

**Figure 2:**
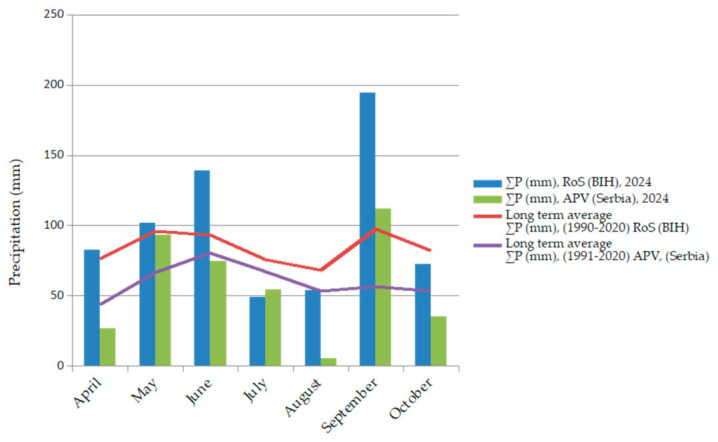
The comparison between the long-term average precipitation (1991–2020) and the precipitation recorded during the April–October period of 2024 in APV and RoS.

Meteorological conditions during the 2024 growing season differed considerably from the long-term climatic averages in both the APV (Serbia) and RoS (BIH) (Figure 2). In APV, the total precipitation during the observation period averaged 57.4 mm/month which was slightly lower than the long-term average of 60.1 mm/month. However, precipitation distribution was highly uneven, with markedly drier conditions in April (26.7 mm vs. 43.9 mm), August (5.2 mm vs. 53.2 mm), and October (35.3 mm vs. 53.3 mm), while May (93.2 mm vs. 66.6 mm) and September (112.0 mm vs. 56.5 mm) were substantially wetter than normal. In contrast, the RoS experienced considerably wetter conditions, with an average monthly precipitation of 99.0 mm compared with the long-term average of 84.2 mm. Particularly high rainfall was recorded in June (138.9 mm vs. 93.4 mm) and September (194.0 mm vs. 97.7 mm), indicating a pronounced excess of moisture during critical crop development and maturation stages.

**Figure 3:**
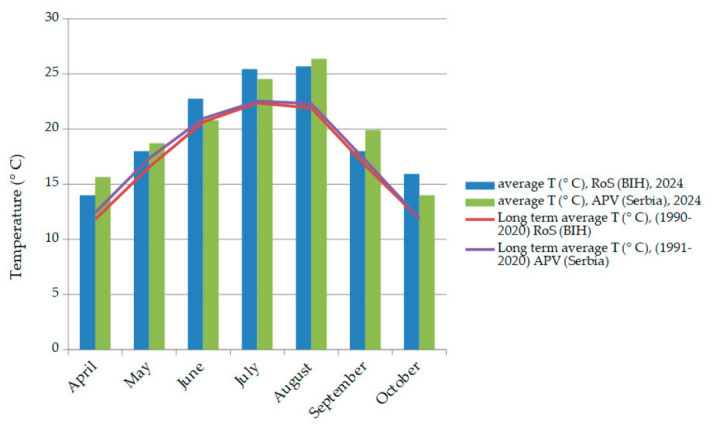
The comparison between the long-term average temperature (1991–2020) and the temperature obtained during the April–October period of 2024 in APV and RoS.

Temperature conditions in both regions were consistently warmer than the historical averages (Figure 3). In APV, the mean growing-season temperature reached 20.0 °C, exceeding the long-term average of 17.8 °C by 2.2 °C, with the largest positive deviations observed in August (+4.1 °C), July (+2.1 °C), and September (+2.7 °C). Similarly, the RoS recorded an average temperature of 20.0 °C compared with the long-term average of 17.4 °C, representing an increase of 2.6 °C. The greatest temperature anomalies occurred during July (+3.0 °C), August (+3.8 °C), and October (+4.0 °C). Overall, the 2024 season was characterized by above-average temperatures in both regions, while precipitation patterns differed substantially, with APV experiencing periods of drought interspersed with intense rainfall events, whereas the Republic of Srpska was generally wetter than normal throughout the growing season.

Overall, the combination of elevated temperatures and reduced rainfall during the late reproductive stages of maize development, particularly the extreme drought recorded in APV during August 2024, created favorable conditions for the occurrence of drought-associated mycotoxins, especially AFs and FBs. In contrast, the higher precipitation levels observed in the RoS during the early growing period may have supported a broader spectrum of fungal development, contributing to differences in mycotoxin profiles between the two regions. Differences between the 2024 growing season and the long-term (1990/1991–2020) climatological averages were evaluated descriptively, as only aggregated reference mean values were available, while full interannual distributions required for formal statistical testing were inaccessible.

### 3.2. Occurrence of Regulated, Non-Regulated and Emerging Mycotoxins in Maize Samples Collected During the 2024 Harvest Season

The LC-MS/MS multi-mycotoxin analysis of 266 maize samples collected during the 2024 harvest season revealed a highly complex contamination profile, characterized by the simultaneous presence of regulated and non-regulated fungal metabolites. Overall, 21 different mycotoxins were investigated, showing considerable variability in occurrence depending on fungal origin and climatic preferences.

Among the regulated mycotoxins, FBs were the most frequently detected contaminants. FB1 and FB2 were present in 81.2% and 51.9% of analysed maize samples, respectively, while the combined occurrence of FBs (FB1 + FB2) reached 88.0%. These findings confirm that fumonisin-producing *Fusarium* species, represented the dominant fungal contributors to maize contamination during the 2024 growing season. The high frequency of FBs may be associated with elevated temperatures during maize flowering and kernel development, since warm and dry conditions followed by periods of humidity are known to favour FB accumulation in maize [36,48].

Aflatoxins were the second most important group of regulated mycotoxins. AFB1, the most toxic and regulated aflatoxin, was detected in 36.1% of samples, while the occurrence of total AFs (AFB1 + AFB2 + AFG1 + AFG2) was 41.4%. The predominance of AFB1 compared with other aflatoxins (AFB2: 2.6%; AFG1: 5.6%) may indicates favourable conditions for *Aspergillus flavus* development. The increased AFs occurrence can be explained by the exceptionally warm and dry summer conditions recorded during 2024, particularly during the reproductive stages of maize development. Heat and drought stress are recognized as key environmental factors increasing plant susceptibility to *Aspergillus* infection and subsequent aflatoxin production [48].

Among the 266 analysed maize samples, OTA was detected in 106 samples, representing 39.8% of the total sample set, which confirms its relevance within the overall mycotoxin contamination profile.

In contrast, regulated *Fusarium* toxins typically associated with cooler and wetter climatic conditions showed considerably lower occurrence. DON and ZEA were detected in only 3.0% and 6.0% of maize samples, respectively. Similarly, type A trichothecenes showed moderate occurrence, with HT-2 toxin detected in 23.7% and T-2 toxin in 18.0% of samples, while their combined occurrence reached 31.6%. These results indicate that the climatic conditions during 2024 were less favourable for DON- and ZEA-producing *Fusarium* species, such as *F. graminearum*, compared with fumonisin-producing species [9,48].

Besides regulated mycotoxins, a high prevalence of emerging fungal metabolites was observed, demonstrating that maize contamination was not limited to compounds currently included in legislation. Among these metabolites, AME showed the highest occurrence and was detected in 86.5% of maize samples, whereas AOH and TEN occurred at much lower frequencies (1.9% and 3.0%, respectively). The high occurrence of AME suggests that *Alternaria* species may represent an important but underestimated contributor to maize contamination under changing environmental conditions.

Moniliformin (MON) was another highly prevalent emerging mycotoxin, detected in 74.4% of samples. The frequent occurrence of MON, together with FBs, suggests the widespread presence of *F. proliferatum* and related species, which are capable of producing multiple metabolites. According to Gruber-Dorninger et al. [16], emerging *Fusarium* metabolites such as MON are increasingly recognized due to their frequent occurrence in cereals, although toxicological data and regulatory limits are still insufficient.

Unexpectedly, EAs were also frequently detected, with at least one EA occurring in 53.4% of maize samples. Ergocristine (ECR) was the predominant compound (40.2%), followed by ergocryptine (ECP; 22.6%), while ergocornine (ECO; 3.4%) and ergosine (ESI; 1.1%) occurred less frequently. Although EAs are traditionally associated with *Claviceps* contamination of small-grain cereals, their detection in maize highlights the advantage of untargeted or broad-spectrum LC-MS/MS multi-mycotoxin approaches for identifying less commonly monitored fungal metabolites.

### 3.3. Descriptive Comparison Between APV and RoS

The occurrence and concentration of major mycotoxins in samples originating from RoS and APV are presented in Table 2. Descriptive statistics revealed substantial variability among samples for most analytes, reflecting heterogeneous contamination patterns. To assess regional differences, ANOVA was applied, and the resulting *F*- and *p*-values were used to identify mycotoxins significantly affected by geographical origin. Although the number of analyzed samples differed between the two regions, both sample sets were sufficiently large to enable robust statistical comparisons. Nevertheless, the unequal sample size should be considered when interpreting regional differences.

The multi-mycotoxin profile of maize harvested in 2024 revealed a high diversity of fungal metabolites in both investigated regions, with distinct differences in contamination patterns between APV, Serbia and the RoS, BIH. These variations were mainly associated with differences in precipitation distribution during the maize growing season (Figure 2), whereas average temperatures were relatively similar between the two regions (Figure 3). Climate-related changes, particularly increased temperatures and altered rainfall patterns, have been recognized as important drivers influencing fungal ecology and mycotoxin profiles in maize [36,48].

Among regulated mycotoxins, AFs, FBs and OTA represented the most important contaminants. AFB1 was detected more frequently in APV (57.0%) than in RoS (26.0%), with higher mean concentrations in APV (25.9 µg/kg) compared with RoS (12.0 µg/kg). Although these differences were not statistically significant (*p* > 0.05), higher AFB1 levels in APV coincided with more pronounced drought conditions during the summer of 2024, which may have contributed to the observed pattern. Similar increases in aflatoxin contamination in Serbian maize during extremely hot and dry years, particularly after the 2012 drought event, were previously reported by Janić Hajnal et al. [33], Kos et al. [35] and Krnjaja et al. [8]. Such conditions favour *Aspergillus flavus* infection and aflatoxin biosynthesis, especially during maize flowering and grain filling stages [36].

Fumonisins were the dominant regulated mycotoxins in both regions. The sum of FBs showed extremely high occurrence (97.6% in APV and 83.4% in RoS), confirming widespread contamination. Comparable mean concentrations between regions indicate that fumonisin-producing *Fusarium* species were highly adapted to the environmental conditions recorded during 2024. Previous studies from Serbia and the Balkan region confirmed that *F. verticillioides* and *F. proliferatum* are the main contributors to fumonisin contamination of maize, with toxin production strongly influenced by temperature and rainfall distribution during silking and kernel development [8,10].

Ochratoxin A (OTA) was detected in 44.7% of maize samples from APV and 37.6% of samples from RoS, with comparable mean concentrations of 13.6 ± 13.4 µg/kg and 14.4 ± 8.5 µg/kg, respectively. No statistically significant differences between the two regions were observed (F = 0.161, *p* = 0.689), indicating that regional climatic differences during the 2024 growing season had only a limited influence on OTA contamination. Unlike sum of AFs, which were strongly associated with the extremely dry conditions in APV, OTA occurrence remained relatively similar in both regions. This finding is consistent with the ecology of ochratoxigenic fungi, primarily *Aspergillus* and *Penicillium* species, which are frequently associated with field conditions [49].

In contrast, DON and ZEA showed considerably lower occurrence. DON was detected in only 3.53% of APV and 2.76% of RoS samples, while ZEA occurred in 0.56% and 8.29% of samples, respectively. These findings suggest that the climatic conditions during 2024 were less favourable for DON- and ZEA-producing species such as *F. graminearum*, which generally prefer cooler temperatures and higher moisture availability [9,48].

Type A trichothecenes showed moderate occurrence, indicating the continuous presence of *Fusarium* species capable of producing T-2 and HT-2 toxins. However, the absence of significant regional differences reflects high variability among individual samples, which is commonly observed in naturally contaminated maize due to heterogeneous fungal development [50].

Besides regulated mycotoxins, emerging fungal metabolites showed high prevalence. MON was frequently detected in both APV (78.8%) and RoS (72.4%), with slightly higher mean concentrations in RoS. The frequent co-occurrence of MON and FBs indicates the presence of multi-toxin-producing *Fusarium* species. Similar weather-dependent variability of MON occurrence in Serbian maize was recently reported by Radić et al. [11], confirming the relevance of MON as an emerging contaminant in this region.

Among *Alternaria* toxins, AME was the predominant metabolite, with high occurrence in APV (92.9%) and RoS (83.4%). The widespread presence of AME indicates that *Alternaria* spp. may represent an underestimated source of maize contamination. Although these metabolites are currently not regulated, their frequent occurrence and potential toxicological relevance have resulted in recommendations for increased monitoring of *Alternaria* toxins in food commodities [16,18].

The detection of EAs in maize further demonstrates the advantage of broad-spectrum LC-MS/MS approaches. Although traditionally associated with small-grain cereals, the presence of EAs in maize contributes to overall exposure and highlights the need to monitor a wider spectrum of fungal metabolites [16].

Large standard deviations relative to mean concentrations indicated pronounced heterogeneity among maize samples, particularly for FBs, MON, and ZEA, suggesting uneven fungal contamination and the presence of highly contaminated individual samples. Although several mycotoxins showed noticeable differences in mean values between APV and RoS, most of these differences were not statistically significant due to high within-region variability and/or the limited number of positive samples.

In general, statistical analysis revealed significant regional differences only for selected fungal metabolites, including STE (*F* = 6.184, *p* < 0.05), TEN (*F* = 55.376, *p* < 0.001), and ergocristine (ECR; *F* = 27.936, *p* < 0.001), with higher mean concentrations observed in RoS compared with APV (Table 2). In contrast, no statistically significant differences were observed for AFB1, DON, FB1, FB2, FUS-X, ZEA, T-2 and HT-2 toxins, MON, AME, OTA, and most individual EAs (*p* > 0.05).

The ANOVA results indicated (Table 2) that the regional factor had a statistically significant effect on the concentrations of sum of AFs, sum of EAs, and sum of T-2/HT-2 toxins, while no significant effect was observed for sum of FBs. Specifically, the concentration of total aflatoxins (sum of AFs) was significantly influenced by the regional factor (*F* = 4.562, *p* = 0.035), indicating that the variability between the compared groups exceeded the within-group variability. Similarly, a highly significant effect was observed for the sum of EAs (*F* = 15.493, *p* < 0.001), suggesting a strong association between the regional factor and the accumulation of EAs. The sum of T-2/HT-2 toxins was also significantly affected (*F* = 4.638, *p* = 0.034), although the magnitude of the effect was lower than that observed for EAs.

In contrast, sum of FBs concentration was not significantly influenced by the regional factor (*F* = 0.023, *p* = 0.879). The extremely low *F*-value and high *p*-value indicate that the differences among groups were negligible relative to the residual variability, suggesting that fumonisin occurrence was largely independent of the examined factor under the conditions of this study.

These findings indicate that despite evident regional trends, such as higher AF occurrence in APV and higher ZEA and MON mean concentrations in RoS, mycotoxin contamination in both regions was primarily characterized by high sample-to-sample variability.

Overall, the results obtained in this study confirm that maize contamination during the weather-stressed 2024 growing season was characterized by complex mixtures of regulated and emerging mycotoxins. Similar observations have been reported globally, indicating that co-occurrence rather than single-mycotoxin contamination represents the predominant scenario in cereal crops [50].

However, a limitation of the present study is that only free forms of regulated and emerging mycotoxins were quantified, while modified mycotoxins, including masked forms, were not assessed. This is particularly relevant for deoxynivalenol-3-glucoside, modified forms of zearalenone such as zearalenone-14-glucoside and zearalenone-14-sulfate, as well as conjugated forms of T-2 and HT-2 toxins, which may occur in maize and contribute to the total mycotoxin burden after hydrolysis during digestion [51,52]. Consequently, the actual contamination level and dietary exposure may have been underestimated. Future multi-mycotoxin monitoring studies should therefore include both free and modified mycotoxins to provide a more comprehensive assessment of contamination and exposure risks.

### 3.4. Compliance with Regulatory Limits

To evaluate the potential safety risk of maize harvested during the 2024 growing season, concentrations of regulated mycotoxins were compared with the maximum levels established by European legislation [53] and corresponding national regulations in Serbia [54] and BIH [55] (Table 3). For maize intended for human consumption, the assessment was performed considering the limits established for unprocessed maize intended for sorting or other physical treatment before consumption, including AFB1 (5 µg/kg), the sum of AFs (10 µg/kg), FBs (FB1 + FB2; 4000 µg/kg), OTA (5 µg/kg), T-2/HT-2 toxins (100 µg/kg), and ZEA (350 µg/kg). Furthermore, for animal feed safety assessment, concentrations of regulated mycotoxins were compared with the maximum permitted and recommended guidance values established by EU legislation [56,57] and corresponding national regulations in Serbia [58] and BIH [59].

These findings confirm that APV maize was more affected by *Aspergillus*-related contamination, most likely due to more pronounced drought stress and reduced precipitation during the summer period. The highest percentage of non-compliant samples was observed for AFs, particularly in APV, reflecting the strong influence of hot and dry conditions during the 2024 growing season. AFB1 concentrations exceeded the EU maximum level of 5 µg/kg established for maize intended for direct human consumption in 36.5% of APV samples, whereas a considerably lower percentage of exceedances was recorded in RoS (12.7%). Similarly, the sum of AFs exceeded the maximum level of 10 µg/kg in 23.5% of APV samples compared with 8.29% of RoS samples. Moreover, according to Directive 2002/32/EC [56] and the Rulebook on Undesirable Substances in Animal Feed of BIH [59], the maximum permitted concentration of AFB1 in maize intended for animal feeding is 20 µg/kg. In the present study, AFB1 concentrations exceeded this limit in 15.3% and 4.97% of maize samples from APV and RoS, respectively. According to Serbian national feed legislation [58], which establishes a maximum permitted level of 30 µg/kg for AFB1 in maize intended for animal feed, exceedances were observed in 12.9% and 1.66% of samples from APV and RoS, respectively. Overall, these results indicate a substantially higher aflatoxin-related risk in APV maize, highlighting the strong influence of regional climatic conditions on *Aspergillus* development and aflatoxin accumulation.

Fumonisins (FBs) represented the second major regulated mycotoxin group regarding compliance with established maximum levels. According to EU legislation [53] and corresponding national regulations in Serbia [54] and BIH [55], the maximum level for the sum of FBs (FB1 + FB2) in unprocessed maize, except maize intended for wet milling, is set at 4000 µg/kg. Although FB1 and FB2 were the most frequently detected mycotoxins in both regions, exceedances of the regulatory limit were considerably lower compared with their occurrence frequency. The percentage of samples exceeding this level was 23.5% in APV and 17.1% in RoS, indicating that FBs contamination represented a relevant safety concern in both regions. However, none of the analysed samples exceeded the guidance value established for maize intended for animal feeding (FB1 + FB2: 60,000 µg/kg), suggesting that despite frequent contamination, the majority of samples remained acceptable for feed use [57,58,59]. The high prevalence of FBs in both regions may indicate favourable conditions for *F. verticillioides* and *F. proliferatum* development. Unlike AFs, fumonisin contamination is often promoted by a combination of warm temperatures and specific moisture conditions during maize silking and kernel development, explaining its high occurrence in both APV and RoS [60,61,62].

Regarding ochratoxin A (OTA), a substantial proportion of maize samples exceeded the maximum level of 5 µg/kg established for unprocessed maize intended for human consumption [53,54,55]. Exceedances were recorded in 44.7% of samples from APV and 33.1% of samples from the RoS, highlighting OTA as one of the major contaminants affecting maize compliance with food safety legislation during the investigated season. None of the analysed samples exceeded the guidance value of 250 µg/kg established for cereals and cereal products intended for animal feeding [57,58,59].

Exceedances of the regulatory level established for the sum of T-2 and HT-2 toxins were infrequent. Concentrations above 100 µg/kg [53,54,55] were found in 3.53% of APV samples and 1.66% of RoS samples, indicating a relatively low risk associated with these toxins. Furthermore, none of the samples exceeded the indicative value for feed use (500 µg/kg) [57,58,59], suggesting a low risk related to these toxins in the investigated season.

ZEA contamination did not represent a significant safety issue in the analysed maize samples. None of the APV samples exceeded the regulatory limit of 350 µg/kg established for maize intended for human consumption after sorting or physical treatment [53,54,55], while exceedance was recorded only in 1.1% of RoS samples. Additionally, all samples were below the guidance value for animal feed (2000 µg/kg) [57,58,59].

Currently, no maximum permitted levels have been established for *Alternaria* toxins in maize intended for food or feed within the European Union, Serbian, or Bosnia and Herzegovina legislation. However, Commission Recommendation (EU) 2022/553 [18] introduced indicative levels for monitoring certain *Alternaria* toxins, including AOH, AME, and tenuazonic acid, highlighting the need for further occurrence data and risk assessment. Although *Alternaria* toxins are not currently regulated, the high occurrence of AME observed in maize samples from both regions indicates that climate-related changes may favor *Alternaria* spp. development and emphasizes the importance of including these emerging mycotoxins in routine monitoring programs.

Unlike regulated mycotoxins, specific maximum levels for EAs have not yet been established in maize matrices under European Union, Serbian, or Bosnia and Herzegovina legislation. Although EAs are traditionally associated with *Claviceps* spp. contamination of small-grain cereals, particularly rye, wheat, barley, and oats [63,64], their detection in maize samples from both APV and RoS in the present study emphasizes the importance of including these non-traditionally monitored metabolites in multi-mycotoxin surveillance programs. The occurrence of ECO, ECR, ECP, and ESI indicates that broader monitoring strategies are needed to better understand changes in mycotoxin patterns, particularly under climate-driven shifts in fungal distribution and contamination risks.

Although regulated mycotoxins, including AFs, FBs, DON, ZEA, and T-2/HT-2 toxins, remain the primary focus of maize safety assessment, the frequent occurrence of non-regulated and emerging fungal metabolites, such as MON, AME, and EAs, observed in this study highlights the importance of expanding routine monitoring strategies. The 2024 growing season, characterized by pronounced heat and drought stress, particularly in APV, was associated with a shift in the mycotoxin contamination profile, with AFB1 representing the main limiting factor for maize utilization according to both food and feed safety criteria. In contrast, exceedances of DON, ZEA, and T-2/HT-2 toxins were rare, whereas the high prevalence of FBs confirmed the continued relevance of monitoring *Fusarium*-related contamination.

Furthermore, the frequent co-occurrence of regulated mycotoxins with emerging metabolites indicates that compliance with current legislation does not fully reflect the complexity of multi-mycotoxin exposure. These findings emphasize the need for integrated LC-MS/MS-based surveillance programs covering both regulated and non-regulated fungal metabolites to improve risk assessment under changing climatic conditions in the Balkan region and beyond.

### 3.5. Co-Occurrence of Investigated Fungal Metabolites

The analysis of multi-mycotoxin contamination revealed a complex co-occurrence pattern of regulated and non-regulated fungal metabolites in maize samples from both investigated regions (Table 4). Multiple mycotoxin contamination was observed in both APV and RoS samples, confirming that maize safety evaluation based only on the occurrence of individual regulated mycotoxins may underestimate the real contamination burden. Similar observations were reported by Eskola et al. [50], who emphasized that simultaneous contamination of agricultural commodities with several fungal metabolites is considerably more frequent than contamination by a single mycotoxin.

In APV maize samples, an average of three regulated mycotoxins and their derivatives were detected per sample, with individual samples containing up to six regulated compounds. Similarly, RoS samples contained on average two regulated mycotoxins, while the maximum number of simultaneously occurring regulated compounds was also six. These results demonstrate that, despite differences in contamination intensity between the two maize-growing regions, both areas were affected by multiple toxigenic fungal species during the 2024 growing season.

Particularly important was the frequent occurrence of non-regulated fungal metabolites. The average number of emerging mycotoxins detected per sample was comparable between the investigated regions (three compounds), with maximum values reaching five in APV and six in RoS. These findings underline the importance of expanding conventional monitoring programs beyond regulated mycotoxins to include emerging fungal metabolites, such as MON, *Alternaria* toxins (especially AME), and EAs. According to Gruber-Dorninger et al. [16], these compounds are increasingly recognized as relevant food and feed contaminants due to their frequent occurrence, limited toxicological information, and absence of established regulatory limits.

Overall, maize samples from both regions showed highly complex contamination patterns. The average total number of detected fungal metabolites per sample was slightly higher in APV (six metabolites) compared with RoS (five metabolites), while the maximum number of co-occurring compounds reached ten in both regions. The higher average contamination complexity observed in APV may be associated with more pronounced drought and heat stress conditions recorded during the 2024 maize-growing season, especially during August. Such environmental conditions may influence fungal ecology, modify the dominance of toxigenic species, and consequently alter mycotoxin profiles, as previously highlighted by Medina et al. [48].

The simultaneous presence of multiple regulated and emerging mycotoxins represents an additional concern because combined exposure may result in additive or synergistic toxic interactions, even when individual mycotoxins occur at concentrations below established regulatory limits. Alassane-Kpembi et al. [65] demonstrated that interactions among mycotoxin mixtures may enhance biological effects compared with exposure to individual toxins, supporting the need for mixture-based risk assessment approaches.

Therefore, the results obtained in this study highlight the necessity of implementing comprehensive LC-MS/MS-based multi-mycotoxin surveillance strategies covering both regulated mycotoxins (AFs, FBs, DON, ZEA, and T-2/HT-2 toxins) and emerging fungal metabolites (MON, AME, and EAs). This approach is particularly important under changing climatic conditions, where shifts in temperature and precipitation patterns may modify fungal populations and increase the complexity of maize contamination.

The complexity of maize contamination was further confirmed by co-occurrence analysis (Table S2). Among 266 analysed maize samples, 189 different combinations of fungal metabolites were identified, demonstrating that multiple contamination rather than single-mycotoxin occurrence represented the dominant contamination scenario during the 2024 harvest season. This finding is in accordance with previous studies indicating that agricultural commodities are commonly contaminated with complex mixtures of fungal metabolites rather than individual mycotoxins [50,65].

The most frequent co-occurrence patterns involved the simultaneous presence of FBs (FB1 and FB2), AME, MON, and FUS-X, frequently accompanied by AFB1 and/or EAs. The frequent association of FBs and MON suggests the important contribution of multi-toxin-producing *Fusarium* species, particularly *Fusarium proliferatum*, which is known for its ability to produce several secondary metabolites, including FBs and MON [11,16]. Furthermore, the high occurrence of AME in these combinations indicates that *Alternaria* species may represent an additional contributor to maize contamination, supporting the need to include *Alternaria* metabolites in extended monitoring programs [16,18].

The simultaneous detection of regulated mycotoxins together with emerging and non-regulated fungal metabolites highlights that conventional monitoring focused only on legislated contaminants may underestimate the real contamination burden and potential health risks. This is particularly relevant because combined exposure to multiple mycotoxins may lead to additive or synergistic toxic effects, even when individual compounds occur at relatively low concentrations [65].

The high diversity of mycotoxin combinations observed in maize harvested in 2024 may also reflect the influence of unusual climatic conditions, particularly elevated temperatures and irregular precipitation patterns, which can modify fungal ecology and shift the dominance of mycotoxin-producing species. Similar climate-driven changes in mycotoxin profiles, including increased occurrence of *Aspergillus*-related toxins and changes in *Fusarium* metabolite patterns, have been previously reported [47,48]. Therefore, the obtained results emphasize the importance of broad-spectrum LC-MS/MS multi-mycotoxin surveillance strategies that include both regulated and emerging contaminants to improve food and feed safety assessment under changing climatic conditions.

## 4. Conclusions

This study provides the first comprehensive report on multi-mycotoxin contamination of maize from BIH, specifically the RoS, and a comparative assessment with APV (Serbia) during the exceptionally hot and dry 2024 growing season.

FBs were the predominant regulated mycotoxins in both regions, whereas the higher occurrence of aflatoxins in APV was associated with more pronounced drought conditions; in contrast, DON and ZEA occurred only sporadically, while OTA showed a similar occurrence in both regions.

The high prevalence of emerging fungal metabolites, particularly AME, MON, and EAs, together with the identification of 189 multi-mycotoxin co-occurrence patterns involving up to ten mycotoxins per sample, highlights the complexity of maize contamination beyond currently regulated mycotoxins.

These findings support the implementation of comprehensive LC-MS/MS-based surveillance programs that include both regulated and emerging fungal metabolites to improve food and feed safety risk assessment in Southeast Europe.

As this study was conducted during a single growing season, the observed relationships should be interpreted as associations rather than causal effects, since mycotoxin contamination is also influenced by other factors, including maize hybrid susceptibility, soil characteristics, and agronomic practices. Therefore, multi-year investigations are needed to confirm the observed trends and support evidence-based monitoring strategies.

## Figures and Tables

**Table 1:** Matrix effect (SSE) and recovery data of the employed analytical method based on the matrix-matched (*R*_E_) calibration curves.

Mycotoxin	Spiking Level (μg/kg) ^a^	SSE (%) ^b^	*R*_E_ (%) ^c^
AFB1	0.3–20	68.9	94.1
AFB2	0.4–10	55.4	79.0
AFG1	0.3–20	59.8	89.0
AFG2	0.8–10	69.2	114
STE	0.1–20	76.5	73.3
DON	4–1750	68.1	104
FB1	0.4–2000	198	99.7
FB2	0.4–2000	142	106
FUS-X	5.0–100	73.0	106
ZEA	0.2–100	63.1	99.2
T-2 toxin	0.1–50	89.3	108
HT-2 toxin	0.1–50	124	102
MON	0.5–100	104	93.2
AOH	1.0–50	84.7	80.0
AME	0.2–20	126	80.9
TEN	0.5–50	76.0	83.4
OTA	0.1–20	105	95.5
ECO	0.2–10	118	79.7
ECR	0.2–10	112	88.4
ECP	0.2–10	113	87.6
ESI	0.2–10	112	89.1

Abbreviations: AF—Aflatoxin; STE—Sterigmatocystin; DON—Deoxynivalenol; FB—Fumonisin; FUS-X—Fusarenon X; ZEA—Zearalenone; MON—Moniliformin; AOH—Alternariol; AME—Alternariol monomethyl ether; TEN—Tentoxin; OTA—Ochratoxin A; ECO—ergocornine; ECR—ergocristine; ECP—ergocryptine; ESI- ergosine. ^a^ Concentration range of analytes for standard, matrix-matched calibration curves and calibration curves of spiked samples (μg/kg); ^b^ SSE-matrix effect (%) calculated by the slope of matrix-matched calibration curve/slope of the solvent calibration curve; ^c^ *R*_E_—Sample preparation recovery (%) calculated by the slope of spiked sample-prepared curve/slope of matrix-matched calibration curve.

**Table 2:** Mycotoxin concentration in maize samples from APV (Serbia) and RoS (BIH).

Mycotoxin	Region	N (%) ^1^	Min–Max (µg/kg) ^2^	Mean ± SD (µg/kg) ^3^	Median (µg/kg) ^4^	Var	*F*	*p*
AFB1	APV	49 (57.0)	0.5522—348	25.9 ± 54.2	6.68	2938	2.701	0.104
	RoS	47 (26.0)	0.7110–129	12.0 ± 21.1	4.53	444		
AFB2	APV	n.d. ^5^	n.d.	n.d.	n.d.		0.000	
	RoS	7 (3.78)	1.43–2.69	1.62 ± 0.4714	1.44	0.222		
AFG1	APV	n.d.	n.d.	n.d.	n.d.		0.000	
	RoS	15 (8.29)	2.22–2.32	2.26 ± 0.0289	2.26	0.0010		
AFG2	APV	n.d.	n.d.	n.d.	n.d.			
	RoS	n.d.	n.d.	n.d.	n.d.			
Sum of AFs	APV	49 (57.0)	0.5522–348	25.9 ± 54.2	6.68	2938	4.562	0.035
	RoS	61 (33.7)	0.7110–131	9.95 ± 19.2	3.02	368		
STE	APV	10 (11.8)	0.5569–2.64	1.26 ± 0.9027	0.6582	0.8150	6.184	0.023
	RoS	10 (5.52)	1.37–2.61	2.10 ± 0.5837	2.51	0.3410		
DON	APV	3 (3.53)	361–531	435 ± 87.1	413	7579	0.135	0.726
	RoS	5 (2.76)	78.1–677	489 ± 237	587	56,285		
FB1	APV	73 (85.9)	2.09–18,149	2270 ± 3438	1089	11,821,950	0.289	0.591
	RoS	143 (79.0)	2.07–29,739	2616 ± 4915	513	24,157,485		
FB2	APV	66 (77.2)	6.10–9059	772 ± 1550	306	2,402,754	2.169	0.143
	RoS	72 (39.8)	3.32–4106	477 ± 666	257	442,914		
Sum of FBs	APV	83 (97.6)	2.09–18,149	2611 ± 3594	1224	12,913,430	0.023	0.879
	RoS	151 (83.4)	2.07–29,739	2705 ± 4951	541	24,515,933		
FUS-X	APV	76 (89.4)	22.8–163	82.8 ± 31.7	83.3	1007	1.035	0.310
	RoS	102 (56.4)	10.1–245	89.3 ± 48.4	83.7	2346		
ZEA	APV	1 (0.56)	n.d.–16.9	16.9	16.9		0.202	0.660
	RoS	15 (8.29)	1.27–706	111 ± 203	13.4	41,188		
T-2 toxin	APV	17 (20.0)	4.00–94.7	19.9 ± 32.4	4.89	1048	1.145	0.290
	RoS	31 (17.1)	0.5294–100	11.3 ± 22.6	2.10	509		
HT-2 toxin	APV	23 (27.1)	0.5192–232	28.9 ± 61.7	3.51	3803	3.352	0.072
	RoS	40 (22.1)	0.5192–93.6	9.70 ± 19.5	1.93	379		
Sum of T-2/HT-2 toxins	APV	25 (29.4)	0.5192–307	40.1 ± 85.6	7.49	7326	4.638	0.034
	RoS	59 (32.6)	0.5192–193	12.5 ± 32.4	1.58	1047		
MON	APV	67 (78.8)	8.78–762	95.8 ± 123	62.7	15,052	1.525	0.218
	RoS	131 (72.4)	7.95–1502	129 ± 204	53.5	41,723		
AOH	APV	4 (4.71)	4.25–30.9	15.5 ± 12.7	13.4	161	4.562	0.122
	RoS	1 (0.56)	n.d.–45.8	45.8	45.8			
AME	APV	79 (92.9)	1.58–861	39.6 ± 125.7	10.6	15,798	2.039	0.155
	RoS	151 (83.4)	2.74–224	24.6 ± 21.3	21.2	455		
TEN	APV	1 (0.56)	n.d.–12.5	12.5	12.5		55.376	<0.001
	RoS	7 (3.87)	15.4–16.7	15.8 ± 0.4181	15.7	0.175		
OTA	APV	38 (44.7)	9.69–78.1	13.6 ± 13.4	10.0	179	0.161	0.689
	RoS	68 (37.6)	0.5510–26.5	14.4 ± 8.50	10.4	72.2		
ECO	APV	n.d.	n.d.	n.d.	n.d.		0.000	
	RoS	9 (4.97)	18.1–30.2	23.2 ± 6.03	18.2	36.4		
ECR	APV	28 (32.9)	1.05–7.27	2.71 ± 1.04	2.65	1.09	27.936	<0.001
	RoS	79 (43.6)	1.00–20.1	11.1 ± 8.40	18.2	70.5		
ECP	APV	14 (16.5)	1.38–20.7	12.8 ± 9.43	20.6	88.9	0.229	0.634
	RoS	46 (25.4)	1.03–18.9	14.0 ± 7.65	18.8	58.5		
ESI	APV	n.d.	n.d.	n.d.	n.d.		0.000	
	RoS	3 (1.66)	1.15–1.62	1.31 ± 0.2677	1.16	0.072		
Sum of EAs	APV	40 (47.1)	1.05–27.9	6.37 ± 7.93	2.29	62.8	15.493	<0.001
	RoS	102 (56.4)	1.02–55.8	17.0 ± 16.3	18.2	267		

APV: Autonomous Province of Vojvodina; RoS: Republic of Srpska; ^1^ N (%): number (percentage) of contaminated samples; ^2^ Min-Max: minimum and maximum concentrations (µg/kg); ^3^ Mean ± SD: mean concentration (µg/kg) ± standard deviation (µg/kg); ^4^ Median: median concentration (µg/kg); ^5^ n.d.: not detected, i.e., below the limit of quantification (LOQ).

**Table 3:** Percentage of non-compliant maize samples intended for human and animal consumption according to Serbian, Bosnian and European Regulations.

	Human Consumption		Animal Feed	
Region	AFB1 ^a,b,c^	AFs ^a,b,c^	AFB1 ^d,g^	AFB1 ^f^
	(>5 µg/kg)	(>10 µg/kg)	(>20 µg/kg)	(>30 µg/kg)
	(%)	(%)	(%)	(%)
APV	36.5	23.5	15.3	12.9
RoS	12.7	8.29	4.97	1.66
	Sum of FB1 + FB2 (>4000 µg/kg) ^a,b,c^		Sum of FB1 + FB2 (>60,000 µg/kg) ^e,f,g^	
	(%)		(%)	
APV	23.5		/	
RoS	17.1		/	
	OTA (>5 µg/kg)		OTA (>250 µg/kg)	
	(%)		(%)	
APV	44.7		/	
RoS	33.1		/	
	Sum of T-2 + HT-2 toxins (>100 µg/kg)		Sum of T-2 + HT-2 toxins (>500 µg/kg)	
	(%)		(%)	
APV	3.53		/	
RoS	1.66		/	
	ZEA (>350 µg/kg)		ZEA (>2000 µg/kg)	
	(%)		(%)	
APV	/		/	
RoS	1.10		/	

^a^ European Commission [53]. ^b^ Ministry of Agriculture, Forestry and Water Management of the Republic of Serbia [54]. ^c^ Council of Ministers of Bosnia and Herzegovina [55]. ^d^ European Parliament and Council [56]. ^e^ European Commission [57]. ^f^ Ministry of Agriculture, Forestry and Water Management of the Republic of Serbia [58]. ^g^ Council of Ministers of Bosnia and Herzegovina [59].

**Table 4:** Average, minimum and maximum number of total, regulated and non-regulated fungal metabolites which co-occurred in maize samples from APV, Serbia and RoS, BIH in 2024.

Region/Fungal Metabolites	APV			RoS		
	N_ave_	N_min_	N_max_	N_ave_	N_min_	N_max_
Number of regulated	3	0	6	2	0	6
Number of non-regulated	3	0	5	3	0	6
Total number	6	0	10	5	0	10

N_ave_—average number of total fungal metabolites represents the sum of average number of regulated mycotoxins and its derivates and average number of non-regulated fungal metabolites; N_min_—minimum number in sigle sample; N_max_—maximum number in single sample.

## Data Availability

The original contributions presented in the study are included in the article/Supplementary Materials; further inquiries can be directed to the corresponding author.

## Supplementary Materials

The following supporting information can be downloaded at: https://www.mdpi.com/article/10.3390/foods15142508/s1, Table S1: Precision data of the employed LC-MS/MS method for detected mycotoxins; Table S2: Co-occurrence patterns of regulated and emerging mycotoxins detected in maize samples collected during the 2024 harvest season from AP Vojvodina (Serbia) and the Republic of Srpska (BIH).

## Abbreviations

The following abbreviations are used in this manuscript:

ACN	Acetonitrile
AF/AFs	Aflatoxin/Aflatoxins
AFB1	Aflatoxin B1
AFB2	Aflatoxin B2
AFG1	Aflatoxin G1
AFG2	Aflatoxin G2
AME	Alternariol monomethyl ether
ANOVA	Analysis of variance
AOH	Alternariol
APV	Autonomous Province of Vojvodina
Arb	Arbitrary units
BIH	Bosnia and Herzegovina
CEN/TR	European Committee for Standardization/Technical Report
CID	Collision-induced dissociation
DON	Deoxynivalenol
EA/EAs	Ergot alkaloid/Ergot alkaloids
ECO	Ergocornine
ECP	Ergocryptine
ECR	Ergocristine
EFSA	European Food Safety Authority
ESI	Ergosine
EU	European Union
FB1	Fumonisin B1
FB2	Fumonisin B2
FBs	Fumonisins
FUS-X	Fusarenon X
GDP	Gross domestic product
HESI	Heated electrospray ionization
HPLC	High-performance liquid chromatography
HT-2	HT-2 toxin
LC-MS/MS	Liquid chromatography–tandem mass spectrometry
LGC	LGC Standards
LOD	Limit of detection
LOQ	Limit of quantification
MA	Massachusetts
MeOH	Methanol
MON	Moniliformin
mTorr	Millitorr
n.d.	Not detected
OTA	Ochratoxin A
PTFE	Polytetrafluoroethylene
R_E_	Extraction recovery
RoS	Republic of Srpska
Rpm	Revolutions per minute
RSDr	Relative standard deviation under repeatability conditions
RSD_R_	Relative standard deviation under within-laboratory reproducibility conditions
S/N	Signal-to-noise ratio
SANTE	Directorate-General for Health and Food Safety analytical quality-control guideline reference
SD	Standard deviation
spp.	Species pluralis
SRM	Selected reaction monitoring
SSE	Signal suppression/enhancement
STE	Sterigmatocystin
T-2	T-2 toxin
TEN	Tentoxin
TSQ	Triple-stage quadrupole/Thermo TSQ instrument series
USA	United States of America
*v*/*v*	Volume/volume
ZEA	Zearalenone
