# Supplementary material for: Weather-Related Variations of Mycotoxins in Maize: A 2024 Study from AP Vojvodina (Serbia) and the Republic of Srpska (Bosnia and Herzegovina)
Source: Foods. 2026 Jul 15;15(14):2508. doi: 10.3390/foods15142508 (PMC13408190; doi:10.3390/foods15142508)
Supplement: Supplementary file 1 [file foods-15-02508-s001.zip › Table S1.pdf]

Table S1. Precision data of the employed LC-MS/MS method for detected mycotoxins.

Mycotoxin	Spiking Level (μg/kg)	RSD (%) (n = 6)[a]	RSDs (%) (n = 3 × 6)[b]
AFB1	0.1 (LOD)*	16.9	20.0
	0.5 (LOQ)**	12.1	15.5
	1	8.91	14.7
	2	6.34	13.2
	10	5.25	10.5
	20	3.69	8.52
AFB2	0.4 (LOD)	14.8	18.6
	1.2 (LOQ)	9.52	12.7
	2.5	6.81	9.35
	5	5.14	7.63
	7.5	4.24	6.45
	10	3.65	5.83
AFG1	0.1	25.5	28.5
	0.5 (LOD)	16.8	19.8
	1 (LOQ)	11.2	15.4
	2	7.82	10.6
	10	4.91	7.13
	20	3.83	5.92
AFG2	0.4	16.2	21.4
	1.2 (LOD)	10.8	15.2
	2.5 (LOQ)	7.43	10.8
	5	5.90	8.54
	7.5	4.84	7.25
	10	4.35	6.52
STE	0.1 (LOD)	14.1	17.0
	0.3 (LOQ)	13.6	14.4
	1	10.3	12.6
	5	9.10	10.1
	10	7.49	9.61
	20	6.30	7.27
DON	4 (LOD)	17.6	19.8
	20	14.7	17.3
	40 (LOQ)	11.3	15.7
	750	5.20	8.16
	1250	4.40	7.78
	1750	3.98	7.63
FB1	0.4 (LOD)	10.9	11.5
	0.8	8.94	9.38
	2 (LOQ)	5.50	5.77
	500	9.28	9.53
	1000	11.3	11.8
	2000	14.6	15.3
FB2	0.4 (LOD)	2.60	2.85
	0.8	2.15	2.53

	2 (LOQ)	3.58	3.79
	500	5.03	6.57
	1000	5.92	6.78
	2000	7.11	8.25
FUS-X	5 (LOD)	16.2	18.6
	10 (LOQ)	13.9	17.1
	20	12.4	14.6
	50	10.1	13.6
	75	9.93	11.2
	100	7.66	9.28
ZEA	0.2 (LOD)	16.3	19.3
	1 (LOQ)	13.6	17.6
	10	12.7	16.8
	20	11.1	13.9
	50	10.7	11.9
	100	5.51	7.20
T-2 toxin	0.1 (LOD)	16.8	19.3
	0.5 (LOQ)	15.8	16.5
	2	14.6	15.0
	10	12.9	13.1
	20	9.75	12.7
	50	8.48	11.0
HT-2 toxin	0.1 (LOD)	16.8	19.4
	0.5 (LOQ)	16.1	18.6
	2	14.5	17.1
	10	12.5	14.9
	20	9.40	13.0
	50	8.14	12.7
MON	0.5 (LOD)	17.9	19.8
	5 (LOQ)	13.9	16.2
	10	12.9	14..4
	20	10.7	12.8
	50	9.66	12.1
	100	3.37	3.97
AOH	1 (LOD)	17.2	19.1
	2 (LOQ)	11.0	12.6
	5	9.21	8.63
	10	7.50	9.21
	20	5.87	6.82
	50	4.83	6.35
AME	0.2 (LOD)	14.3	16.9
	0.5 (LOQ)	12.9	13.3
	2	8.97	10.2
	5	5.64	7.35
	10	4.54	5.19
	20	3.50	4.04
TEN	0.5 (LOD)	17.3	19.2
	1 (LOQ)	14.6	17.3
	2	12.1	13.4

	10	10.6	11.7
	20	7.35	10.0
	50	5.86	6.81
OTA	0.1 (LOD)	14.1	14.9
	0.5 (LOQ)	10.2	12.7
	3	9.43	11.9
	5	7.54	9.84
	10	6.90	8.02
	20	5.83	6.90
ECO	0.2 (LOD)	18.1	19.9
	0.4	17.1	19.3
	1 (LOQ)	15.8	16.9
	2	13.6	15.5
	5	11.5	14.5
	10	6.91	8.29
ECR	0.2 (LOD)	17.6	19.4
	0.4	14.1	16.9
	1 (LOQ)	11.5	14.0
	2	9.46	12.8
	5	8.82	11.8
	10	8.68	9.26
ECP	0.2 (LOD)	15.9	18.9
	0.4	14.9	18.1
	1 (LOQ)	10.2	11.6
	2	9.52	8.18
	5	7.33	8.00
	10	6.30	7.42
ESI	0.2 (LOD)	16.4	18.1
	0.4 (LOQ)	13.4	17.4
	0.8	12.1	14.2
	2	10.9	11.8
	5	8.22	10.5
	10	6.66	7.12

Abbreviations: AF – Aflatoxin; STE – Sterigmatocystin; DON – Deoxynivalenol; FB – Fumonisin; FUS-X - Fusarenon X; ZEA – Zearalenone; MON – Moniliformin; AOH – Alternariol; AME – Alternariol monomethyl ether; TEN – Tentoxin; OTA - Ochratoxin A; ECO – ergocornine; ECR – ergocristine; ECP – ergocryptine; ESI-ergosine. *LOD—limit of detection; **LOQ—limit of quantification; [a]RSD (%)—relative standard deviation of 6 replicates at six concentration levels using the spiked blank maize flour and the matrix-matched calibration (MMC) curve; [b]RSDs (%)—relative standard deviation of 6 replicates at six concentration levels using the spiked blank maize flour and the MMC curve, over the course of three days, using the same instrument and by the same operators.
